# Health Food Ad

**Published:** 1902-11

**Authors:** 


### Health Food Ad.

The day of his execution had come.

He arose, dressed himself and performed his ablutions.

"What will you like for breakfast?" asked the kind-hearted jailer.

"I suppose you have ham and eggs, fried potatoes and coffee?" said the condemned man.

"Yes," replied the jailer.

"Well. I don't want them," he rejoined, with a discordant laugh. "Bring me some kind of health food and a cup of cereal coffee, and I will die with joy."—*Chicago Tribune.*

